# Easy Methods of Detecting Blood-stains

**Published:** 1880-11

**Authors:** D. S. Kellicott

**Affiliations:** Buffalo State Normal School


### EASY METHODS OF DETECTING BLOOD-STAINS.

BY PROF. D. S. KELLICOTT, BUFFALO STATE NORMAL SCHOOL.

THE detection of blood-stains, in a medico-legal sense and for other purposes, is certainly a question of grave importance. In criminal cases experts are ordinarily employed, but it is a hard fact that these experts have various degrees of expertness. Physicians are often called upon to testify in court, either as experts, or to corroborate or destroy expert testimony; therefore, the plain obligation to acquaint themselves with the recognized methods, and the state of scientific and professional knowledge relating to this subject, and to get both a theoretical and practical acquaintance with these methods. Although I am neither a doctor nor a professional chemist, I undertake to state several easy methods of detecting blood-stains, and in your presence attempt to demonstrate their efficiency.

The questions presented are these, "Is a given spot or stain caused by blood, and if so, is it human or not? two very distinct inquiries. To the first the examiner can ordinarily give a positive answer, obtaining his proof from one or all of these methods 1. Chemical reactions. 2. Microscopic examinations. 3. Spectroscopic examination.

It is often of much consequence to be able to affirm that the spot in question is due to blood. To the second question, although of more consequence, unfortunately a less positive answer can be given. There is at present but one known method of approaching the problem, i. e., by the size, shape and character of the corpuscles under the microscope, and when these are restored from blood once dried, many, I may say most, experts deny that these qualities are sufficiently constant to be decisive. The chemical and spectroscopic behavior of red blood after drying, derived from whatever source, is now held to be identical. I shall omit all consideration of the spectroscope in relation to this matter, except to remind you of Beal's statement that "the application of the microspectroscope to the detection of blood-stains has, on the whole, been most satisfactory; probably for the future this test will be that universally adopted." (*Mic. in Med.*) On the other hand Dr. J. G. Richardson says, "That it is not so delicate as the microscope and of no use in determining the kind." (*Med. Mic.*) I have too little practical acquaintance with this instrument to have an opinion. It seems to me that the microscope or chemical tests are sufficiently delicate for most cases; besides I incline to the plainest methods, yet after all, the nature of the case must determine what means shall be employed.

The easy chemical tests may be mentioned as follows:

1. Blood readily dissolves in distilled water, giving a reddish color. Blood dried upon iron and acted upon by the hydrated oxide of iron is insoluble in water. It dissolves, however, on adding citric acid. The undissolved threads of fibrine may be examined under the miscroscope and chemically tested. (Dissolves in acetic acid, colored by test liquid of Millon and Pettenkofer).

2. To the solution in water add weak ammonia, no change of color, or at least no change to *crimson*, which excludes certain vegetable stains.

3. Boil a small quantity of the aqueous solution, the color is discharged and grayish floculi appear in it from coagulated albumen; these disappear on adding solution of caustic potassa, the color becoming green by reflected light, red by transmitted light.

4. Obtain the hæmin crystals. This last reaction I consider very delicate, the crystals never failing to appear even when the quantity of dried clot is exceeding small; a fragment large enough to see distinctly is ample to afford scores of these characteristic forms. There are several methods of obtaining them, viz.:

1. Virchow's plan: The fragment is crushed, an equal quantity of common salt added. After thoroughly mixing, moistened with glacial acetic acid, covered and brought to boiling, or to dryness over the water bath.

2. Crush the fragment, add a drop of water holding a trace of common salt. I use one drop of a one-per-cent. solution to ten of water. Cover and so place a drop of glacial acetic that it will slowly run under, then heat over the water bath as before.

3. Boil the powdered blood with glacial acetic acid in a test tube and evaporate to dryness on a glass slip.

Other courses are prescribed, but unless the case is a special one, these, while simple, are entirely satisfactory. Reasonably skillful manipulation can not fail to give the charateristic, brown rhomboidal crystals, many lying singly, others in crosses or stars. There are no known crystals of other substances thus obtained likely to deceive a practiced eye. Different kinds of blood give similar crystals. They may be examined with a quarter. I prefer the first method named above, although the second gives the crystals more evenly distributed over the field. [Here the foregoing reactions were shown and the Association took an informal recess in order to examine the prepared crystals.]

The microscopical examination of blood-stains has received much careful attention, and this instrument of precision has in this field undoubtedly great possibilities; it has surely suffered on account of its friends, still I am not without hope, indeed I have the expectation that it may yet abundantly triumph. Is it not so that adverse opinions, as to its ability in these matters, arise more often from want of excellence in the glasses, or from want of manipulative skill in the operator than from the nature of the case?

The microscopic test depends mainly upon obtaining the corpuscles, red or white, or both; to determine whether or not it is mammalian upon their shape, size, melus, and if mammalian whether it is human or not, by the size alone. There are several rules given us for restoring the corpuscles; the simplest and most rational are these:

1. Dr. J. G. Richardson's method: Crush a minute fragment under cover, focus upon a suitable piece, a thin edge, then slowly run under distilled water.

2. The same with a one-per-cent. solution of common salt.

3. The same with glycerine and waters—specific gravity 1028, prepared by mixing four fluid ounces of water with three and one-half drachms of glycerine; some carbolic acid should be added if the mixture is to be kept for use.

After using each of the above fluids, I conclude that distilled water is quite as sure to give results as the others. To obtain red blood globules from a dried mass, sufficiently perfect for the determination of their characters, is not nearly so easy or sure, as the books make it, and to get them so well that measurements differentiate the blood is of course much more so. Beal and Richardson mention high powers and glasses of superior quality as the necessary equipment for such work. My experience teaches that there is no use to try to do anything with this matter without high powers. I believe the very best objectives for the purpose are the modern four-system, sixth or tenth of the highest angle, and these in connection with solid eye-pieces.

Experimenting with human blood, dried in a mass on a glass slip, using a fairly good one-sixteenth inch objective, I obtained results as follows: 1. Every trial gave the lymph globules. 2. Every trial gave, after the color was well discharged, threads of fibrine. 3. One-half of the trials gave the red globules so well that I could declare that they were not from reptiles, fishes, or birds. 4. One attempt in five gave the red ones so satisfactorily, that some few could be measured with accuracy, and by the way, my measurements of such (using the camera) came very near the standard size of these discs when dried in film on a glass slide. A series of experiments with dried snake's blood, using the one-eighth inch objective, gave rather more favorable results. When too much water was used, and the outline of the corpuscles were invisible, the nuclei were often seen in masses which were quite deceiving, under a quarter inch objective they appeared quite like globules of another sort.

Is a stain due to human blood or not? I would not undertake to say in a case where any thing in particular depended upon it. I will not say that I think it doubtful whether those professing to be able to so determine as really able or not, but I can pretty safely say that the number who ought to attempt such determination are very few. [Here slides were prepared, and the globules exhibited under the microscope.]

